# Conceptualizing a less paranoid schizophrenia

**DOI:** 10.1186/s13010-023-00142-8

**Published:** 2023-11-08

**Authors:** James Long, Rachel Hull

**Affiliations:** 1 Department of Psychology, Chestnut Hill College, 7113 Valley Avenue, Philadelphia, PA 19128 USA; 2 Chestnut Hill College Department of Professional Psychology, 9601 Germantown Avenue, Philadelphia, PA 19118 USA

**Keywords:** Schizophrenia, Schizophrenia spectrum disorders, Schizotypy, Schizoid, Psychosis

## Abstract

Schizophrenia stands as one of the most studied and storied disorders in the history of clinical psychology; however, it remains a nexus of conflicting and competing conceptualizations. Patients endure great stigma, poor treatment outcomes, and condemnatory prognosis. Current conceptualizations suffer from unstable categorical borders, heterogeneity in presentation, outcome and etiology, and holes in etiological models. Taken in aggregate, research and clinical experience indicate that the class of psychopathologies oriented toward schizophrenia are best understood as spectra of phenomenological, cognitive, and behavioral modalities. These apparently taxonomic expressions are rooted in normal human personality traits as described in both psychodynamic and Five Factor personality models, and more accurately represent explicable distress reactions to biopsychosocial stress and trauma. Current categorical approaches are internally hampered by axiomatic bias and systemic inertia rooted in the foundational history of psychological inquiry; however, when such axioms are schematically decentralized, convergent cross-disciplinary evidence outlines a more robust explanatory construct. By reconceptualizing these disorders under a dimensional and cybernetic model, the aforementioned issues of instability and inaccuracy may be resolved, while simultaneously opening avenues for both early detection and intervention, as well as for more targeted and effective treatment approaches.

## Background

Schizophrenia is one of the oldest and most studied mental disorders within the history of psychological science. Mental and medical health practices consistently fall short for patients diagnosed with schizophrenia spectrum conditions. Treatment plans are predominantly stereotyped, heavily reliant on second-generation antipsychotics [1–4], and rarely include validated psychosocial or psychotherapeutic interventions [5–7], Verdoux et al., 2010). This pattern persists despite widespread agreement on heterogenous presentation and treatment outcome [3, 8–11], moreover, a tremendous amount of the variability in prognosis, etiology, and even the effectiveness of pharmacotherapy is accounted for by factors overlooked in diagnosis and/or outcomes monitoring [10, 12–14]. This poses an ethical issue in that, to the extent that the goal of the mental health field is to alleviate human suffering and to promote human flourishing, past and present approaches miss the mark. This also implies that the currently accepted conceptualization(s) of schizophrenia spectrum disorders is at least partially flawed. Finally, this treatment failure incurs a heavy social and economic cost. The US domestic economic cost of schizophrenia is staggering, estimated at US$60 billion per annum [15], moreover, accounting for indirect costs, this is likely a conservative estimate.

The reasons for this systemic deficiency are as diverse as the schizotypy spectrum itself, ranging from foundational flaws in the current diagnostic model of mental health to constraints inherent in the contemporary mental health system. The increase in biologization of the field [16] has encouraged efforts to identify neural correlates in line with a disease model, while the preponderance of evidence indicates that schizotypy, in all of its manifestations, is profoundly moderated by environment. Such emphasis on materialism has not mitigated stigma or improved treatment outcomes and likely contributes to the current and historical plight of the population [5]. Accounting for the schizotypal population as a whole, it may be appropriate to conceptualize schizophrenia as “the story of the way that poverty, violence, and being on the wrong side of power drive us mad” ([17], p.197), or perhaps more succinctly, “bad things happen and can drive you crazy” ([3], p. 145).

Housing the etiological locus solidly within the realm of environment and not genetics is consistent with the Hearing Voices Movement (HVM; [18], which seeks to reframe psychosis symptoms within culture and context, and refrain from treatment of those experiences as a “biogenetic disease state,” [18], p. 134) which is not supported by recent data. While the model and perspective proposed in this paper aspire towards a new operational explanation, the body of research necessary and sufficient for such a definition is either nascent or theoretical,nonetheless, there exists precedent for de-stigmatizing and de-pathologizing psychosis (the symptom) and schizotypy (the syndrome).

The heterogeneity of presentation and treatment outcome has lead other researchers to argue that the construct of schizophrenia as a whole needs to be reconceptualized [3, 18–22]. Inasmuch as disagreement may be attributed to the unknowns inherent in any scientific dispute, the issues around schizophrenia are more pronounced. This is likely due to numerous factors. The foremost being that all issues involving psychosis lie contrary to the unspoken morality of post-enlightenment societies, the overarching dogma of which describes a world which is ultimately without contradiction, and which can only be truly understood through the mechanisms of reason. The second unspoken assumption is that such an understanding is unquestionably good. Given the ubiquity of instrumental rationality as final arbiter of value, the ability to participate in consensus reality becomes a measure of one’s human worth, and one’s divergence from said explanatory consensus is an index of one’s illness. This cultural and methodological axiom thus doubly binds the schizotype, as their phenomenological position is simultaneously given an unspoken moral dimension while providing tools for study and care predisposed to pathologize and dispense with said position as inherently aberrant and symptomatic.

The present article will begin with rationale explicating the historical, philosophical, and conceptual difficulties precipitating myopic approaches to treatment and research within the population. Secondly, it will survey relevant evidence from various disciplines which provide converging evidence toward a more comprehensive view of schizotypy and its manifestations with psychotic features. Finally, the present authors propose a more thorough conceptualization of the schizotypal spectrum, along with a framework to reconceptualize schizotypal diagnoses as identifiable patterns within a dimensional paradigm incorporating both “healthy” and “pathological” members. Finally, clinical implications will be discussed.

Ultimately, it will be argued that a properly developed cybernetic model, rooted in trauma-informed personality theory, best captures the nature and breadth of this human experience. It is the present authors’ hope that with such a reconceptualization, self-stigma in those with schizophrenia will mitigate as well, as self-stigma in individuals on the schizotypal spectrum has been recognized as a “second illness” [23], as cited in [3, 24–29].

## Historical and philosophical precedents

### Underlying axioms and paradigmatic blind spots

Although there is room to debate many of his specific points and inferences, Foucault (1965) addressed many of the genealogical ideas that underlie current mental health practice. Salient are the social and moral implications of mental illness in a post-enlightenment age. Reason and empiricism were held up as the means by which humanity would extract itself from the arbitrary and oppressive moral and social systems that characterized the preceding epochs. However, as no human or society can exist without an orienting value system (ought from is) [30], this revolution merely altered the parameters. Moral punishment became reserved for “healthy” individuals with deviant behavior, while those whose behavior was determined to be medical in origin were to be cured (i.e. brought back to reason and regulated passions). However, in both cases it was deviance from the collectively understood “good” which was targeted. In the former case, deviant behavior was punished or corrected through learning, while in the latter psychological deviance imbued society with a moral duty to treat or cure. Much of our current approach to treatment, such as cognitive-behavioral theory, is predicated on this idea, that it is irrational thoughts that cause distress, based on the underlying assumption that showing the person that their thinking is irrational is itself a kind of cure for their experience.

In a recent review of 30 consecutive court-mandated medication hearings, 29 were approved without a jury, most in cases where serious neurological damage had been caused by medications and at higher doses than would be recommended. All cases of treatment had entirely discounted options such as psychotherapy, despite defendant and family protest, demonstrating that the line between moral capability, medical impairment, and societal responsibility is still quite blurry [5].

The presenting issues involving psychosis are inherently aberrant against the axioms of the age, giving their expression a numinous quality absent from comparable symptomology. While a “healthy” person can empathize with a depressive or obsessional person (seeing their experience as merely an extreme version of their own), the hallucinations, delusions, disorganized thoughts, and behaviors of psychosis are deeply unsettling. Such a person may be pitied or sympathized with, but how can one empathize with a person who is not participating in consensus reality, let alone take their perspective seriously?

The rational-empirical model Foucault was dissecting had more comprehensive effects as well, establishing the parameters by which reality was defined. Truth was to be determined through careful observation, data collection, and objective analysis of results. One could subsequently remove the confounds of arbitrary values and subjectivity and determine what was and was not “real.” This idea has been one of the most important and useful tools in human history, and its benefits cannot be overstated; however, as with any idea, it rests upon axioms and results in outcomes with predictable constraints.

As much of the unspoken paradigm scaffolding within scientific models can be traced back to the ideas of rationalism and empiricism, and to the extent this paper aims to address axiomatic flaws in those models, it is worth exploring the concepts. Broadly speaking, the rationalists argued that knowledge was attained through logic and reason and that human understanding was founded on innate ideas. In contrast, the empiricists argued that humans were tabula rasa, and that knowledge was gained exclusively through sensory experience. Responding to both, Kant [31] outlined his synthetic a priori propositions on reason and its recognition of necessary cognitive structures preceding sensory modification. He argued that the only way in which a human being could have knowledge in a functionally infinite sea of data was through categorization. That we contained an innate scaffolding which predisposed us to select and judge our sensory data, and that without such we could not possibly perceive the world in any meaningful way at all. Moreover, as we were goal-oriented creatures, this was an inherently value-laden conceptualization. While this perspective was revolutionary in overall enlightenment thinking, its ultimately phenomenological approach to reality has had little impact on the scientific endeavor to understand that very rationality.

What has been retained is something of the desire which drove Kant’s deductive exploration; namely to arrive at a surety of knowledge without call to a divine authority. His endeavors into synthetic a priori knowledge assumed and ultimately sought to prove that foundational sure knowledge could be found and worked upwards from to arrive at universal and objective truth. The distillation of this is the reductionism which subtly underlies scientific inquiry to this day; the belief that the best way to understand a complex system is to break it into its smallest constituent units and then extrapolate upwards. From this we may identify the roots of the atomism and materialism which will be discussed further in this paper.

Equally important to these methodological axioms however is the subtle implications of a unitary truth when applied to the psyche and its inquires (i.e. that a single correct perspective is attainable and thus deviation therefrom is an error to be corrected). Through this lineage one has dispensed with subjectivity (the valuing ‘ought’ and its many constraints) in favor of objective proxies as a means of study and have thus over time, often with a sense of moral duty, come to see subjectivity as (at best) epiphenomenal. This trend has likely reinforced, if not outright caused, the current preference for biological interventions for subjective experiences while viewing aberrant subjectivities as a problem to be solved. The more strictly empirical approach can likely be attributed to the structural disconnect between verifiable data and subjective experience endemic to objective and materialistic approach to science; however, this inattention has created potential blind-spots in investigative assumptions.

### Congruent models and fractal patterns

While Kant arrived at his categorical phenomenological conclusions from deduction and intuition, Jean Piaget [32] outlined a nearly identical process by observing the developmental construction of schemata in young children. In highlighting the evolution of children’s reports and understandings of reality, Piaget stands as a kind of naturalist of the same process proposed by Kant, in which a rather simple set of structures give rise over time to the operations of intelligence. In both Kant’s concept of understanding and Piaget’s concept of a schema (in a total sense), a person can only conceive of realities congruent with the structure of their framework,that is, they have a subjective perspective. Where Piaget’s observations stand out is in his detailing of the process by which these structures update and organize themselves across time (e.g. the famous cognitive revolution children experience when developing a theory of mind). In light of contemporary evolutionary, genetic, and personality research though, the full scope of potential schematic diversity remains an open question. That is, to what degree do we converge upon a universal perception of reality and to what degree are we perceiving and conceptualizing differently?

Regardless, the implication of the symptom-focused taxonomy of the DSM or ICD is that subjective diversity is either irrelevant or non-existent and thus our scientific study of the mind cannot account for it. Given that our schemata expand and evolve through assimilation and accommodation, and that they are the medium through which we construct our reality, the means by which that dynamic equilibrium is affected by biological predisposition and the specific nature of events experienced during this process must be accounted for if psychology is to succeed as a scientific pursuit. Understanding the forces which led to and maintain such an equilibrated structure carries implications for psychological models of cognition and perception as well as for clinical treatment.

Symmetrical to Piaget’s schemata, Kuhn’s [33] description of the paradigmatic nature of scientific discovery demonstrates this process at the level of consensus reality and may also indicate the core problem with current models of schizophrenia-spectrum disorders. Much in the same way a developing child in Piaget’s model works pragmatically within a ‘good enough’ schema until sufficient development and anomalous information induces a reorganizational (and thus perceptual) classification, Kuhn noted that scientific paradigms (collective schemata of interpretation and behavior) progress until sufficient anomalous data induces a reorganization of core assumptions, such that previous evidence and anomalous evidence remain, are accounted for, and explained. Much as two individuals with incompatible schemata would find mutual understanding impossible without accommodation on one or both ends, the current paradigms informing much of psychological research (e.g. objectivism, materialism, etc.) cannot account for the symptomatic manifestations of the schizotypal spectrum other than as pathological deviation, and thus remain obtusely focused. Instead, models must account for the biological and environmental impacts on schematic development as a necessary component in any defensible definition of mental health. Currently, models such as the Five-Factor Model of personality (FFM); [34] offer avenues to begin exploring and discussing this scientifically, however their implications have yet to meaningfully propagate throughout the psychological field.

### Outcomes and limitations of unexamined axioms

In a discussion on the theoretical challenges facing psychology today, Slife (as cited in Lambert, 2004) identified numerous constraints on theory and its practical application. Objectivism essentially posits that the logic inherent in the methods and techniques of science and clinical practice can be relatively free of systematic biases and values. This is achieved through the use of logical reasoning (rationalism) and unbiased observation of phenomena (empiricism). This permits a certain unbiased standard of proof that can be verified and agreed upon without appeal to arbitrary authority or preconceived assumptions; however, it also leads to limitations. It bounds what can be studied (and thus proven) to those things which can be observed and replicated. In psychology, this translates to examining the psyche by proxy. As one can have no direct observation of experience, various behaviors are examined instead with the assumption that these act as indicators of internal states and dynamics. Where behaviors cannot be determined, states are operationalized; anxiety becomes the nexus of racing thoughts, restlessness, distractibility, etc. What cannot be captured is the valence, meaning, and experience of anxiety, or the idiosyncratic relationships an individual's anxiety has with their own history, conceptual framework, and day-to-day experience.

For example, within a psychodynamic framework, a clinician would find it important to determine whether a patient’s depression was anaclitic or introjective, thus accounting for the inner subjectivity underlying the overall state. However, most strictly empirical research and certainly most pharmacological research must measure itself by symptom reduction within the DSM criteria of depression, which does not account for personality style. In this way, the methods by which research is conducted systemically deem irrelevant domains of human experience and psychological evolution, becoming blind to them.

A second axiom is materialism, which posits that psychological experiences will eventually be shown to have observable and biological bases. All psychology is simultaneously biology. As with objectivism, this assumption is predominantly benign or beneficial; however, it too creates complications. One is the implicit causal direction; that the core problem is contained within, and thus solvable and explicable through, biology. Indeed, materialism is tightly wedded to objectivism as it is often far easier to study physical systems than social or psychological ones. This belief underlies much of the faith in and reliance on pharmaceuticals as “cures” for psychological disorders. Once a biological correlation is identified, it is treated as the cause despite our knowledge that the relationship is more complicated; moreover, the entire DSM/ICD classification systems assume biologic etiology. This causal direction also promotes an aura of preeminence to biological markers over holistic biopsychosocial assessment. For example, if research finds that serotonin differences act as a biomarker of depression, it is assumed that such differences causally precede the psychological state and so become the target of treatment, despite evidence that such neurotransmitters are themselves greatly influenced by environment and cognitive framing [35, 36].

Finally, the axiom of atomism assumes that the qualities of people are contained within the individual, and so treatment should focus on individual cognition, biology, and behavior. As can be seen, atomism dovetails seamlessly with reductionism (the individual is the indivisible member of the collective), materialism (the biological operations of others do not influence those around them), objectivism (it is easiest to observe the components of an individual rather than the network of influences between them and their environment over time) and implicit morality (the locus of choice and thus moral human agency exists within the individual). These assumptions, while almost certainly a necessary heuristic, can lead researchers and practitioners to underestimate or ignore the impact of relationship factors or social context.

These and other axioms inform many more areas of human life than the field of psychology, and in many of them (e.g. particle physics), they may operate more or less perfectly. Inasmuch as psychology is to be the study of the psyche, however, it must at some point include the study of human subjectivity; moreover, it is the experience of suffering that we aim to alleviate, not its proxies. This is decidedly difficult within a framework that goes to great lengths to remove all subjectivity before even beginning its search.

Furthermore, whatever it is one means by the psyche, it is a dynamic and multi-level phenomenon. It is shaped by the past through memories and biological alterations (processes which continue to change throughout the lifetime); simultaneously, how a person conceives of the future continuously alters behaviors, cognitions, and relationships (which in turn recontextualizes memories and alters biology). Each of these is further informed by the idiosyncratic relationships a person has (as well as how he conceives of them) and the environment in which he lives (physical and social). It is in fact this entire set of inter-penetrating and interlocking systems which determine how any particular experience manifests. Within the framework above, those elements which are most difficult to operationalize or which lie perpendicular to accepted rigor are granted a reality significantly less substantial than those which are considered more “evidence-based,” and thus most often lay unaccounted for in final etiology and nosology.

The DSM’s taxonomy, as well as its preeminence in mental health practice, is the distillation of this process. It testifies to the strengths this approach has brought to the field, and simultaneously contains its weaknesses. As has been noted by clinicians throughout its development and subsequent iterations, the DSM’s approach dispenses with, misinterprets, or lies contrary to the bulk of historical and contemporary clinical wisdom [37–40].

Without an explicit definition or discussion of mental health, the DSM implies that the removal or reduction in stated symptoms is the goal (empiricism and reductionism). This creates the following three issues: (1) While symptom relief may be desired, no other medical professional would equate symptom reduction with a cure. (2) The DSM can offer no discussion or guidance on important qualia within those symptoms (e.g. recall objectivism—is the depressive experience fundamentally anaclitic or introjective). (3) The DSM offers no insight on the depth of, interactions between, or potential functions of experiences as outlined. One may contend that none of these was meant to be the function of the DSM, which was instead intended to be one tool in an arsenal the clinician would bring to bear. However, whether due to the constraints of time and energy, the demands of insurance companies, the limitations of training, the (above-outlined) biased nature of research, the fact that the DSM is subservient to the ICD, or any combination of these factors, it is often the case that the DSM is used in exactly this manner. In 2013, the National Institute of Mental Health (NIMH) ceased funding DSM-based research citing the model’s overall “lack of validity” (Insel, as cited in [41], p. 522).

The weight of evidence accrued even within this framework calls its assumptions into question. It has been noted, for example, that in clinical settings, depression is usually paired with anxiety and somatic symptoms, while also manifesting in highly variable ways (aggression, risk-taking behaviors, etc.) [39]. Simple diagnosis is insufficient for treatment planning [42] and ultimately leads to stereotyped and imprecise responses; moreover, if disorders were, in fact, distinct categories, one should expect them to have distinct boundaries with matched biological correlations. However, antidepressant medications are used to treat anxiety and other mood disorders, and antipsychotic agents are prescribed for bipolar disorder and various severe personality disorders. Symmetrically on the treatment side, cognitive-behavioral therapy (developed to address internalizing disorders) has since expanded to encompass nearly every class of mental disorder [43], despite major methodological flaws in the research that supports CBT [44]. Moreover, longitudinal and epidemiological evidence indicates that etiology, prognosis, and even pharmaceutical effectiveness depend on psychosocial factors disregarded in the current taxonomy. For example, in patients with schizotypy and psychosis, treatment and pharmacological outcomes depend much more on factors such as childhood trauma, social factors, and neurocognition [3, 11, 45].

While the full impact of this dynamic stretches throughout the whole of psychology, select points are particularly relevant. In striving toward the objectivity so highly valued under our society's ruling metanarrative, psychological practice and research has adopted, almost axiomatically, a “disease” model of psychological suffering; we document symptoms and attempt to place them into distinct categories which would have distinct biological/behavioral underpinnings which can be discretely addressed. Thus, the *experience* of a disorder and its treatment can be generalized and divided into discrete components. It also implies that, as the symptom expressor, the problem lies within the individual and so must be addressed at that level. As was outlined through underlying Enlightenment philosophies earlier, these assumptions are natural outcomes; that the moral locus lies within the individual’s own rationality (their claim to full personhood) and so remains unquestioned within most research models. As Higgs [18] purported, “the advancement of neoliberal values and policies likewise favors naturalizing inequality through the lens of biology, locating the suffering caused by *social* problems within *individual* bodies, which are perceived as self-contained and (ideally) fully independent” (p. 138).

Given that such a model is conducive to research, conforms well to the needs of insurance and pharmaceutical companies, and carries the implied authority of decades of acceptance, the situation is self-reinforcing. Moreover, the taxonomic model implies a baseline human experience, deviation from which constitutes the nature of psychopathology. Given biologization and atomism, the problem is seen as localized within the person, and treatment focuses on the adjustment of biological systems and the resolution of irrational thoughts and behaviors. In so doing, the complexity of human psychology and the entirety of subjectivity is done away with; a serious issue given that the psyche is defined by and experienced through subjectivity. Indeed, “a grisly tradition of ‘biologizing social facts’ exists within psychiatry” [18], p. 137–138). This divides much of psychology as a body of knowledge from the bulk of clinical wisdom and makes the training, expansion, and transmission of this understanding difficult at best. It limits the field’s understanding of human psychology and our ability to address individuals’ actual experiences. For example, in addition to the model’s inability to discuss characterological differences or dynamic interactions between disorders and psyche, it has nothing to say about the positive side of human experience as a necessary component of health. Finally, while this complex is problematic for any psychological disorder, it becomes more so the more deeply and/or longitudinally it exists within the client, and the further from placidity, conformity, and rationality it takes her. A person experiencing an anxiety attack has a problem, a person with borderline personality disorder needs extensive management, and a person with schizophrenia is beyond the pale.

### Philosophical summary and subsequent steps

The broad philosophical assumptions which form the basis for the rational-empirical model informing current scientific inquiry have given primacy to objectivity as the measure of truth as a matter of course. In so doing, it has ultimately directed research and our collective understanding of psychology into a taxonomic and symptom-based structure which will naturally prioritize biological causation and atomistic approaches to treatment. Simultaneously, the same axioms which dictate our current scientific paradigm contain implicit moral assumptions which reflexively pathologize experiences, perspectives, and expressions which are deemed “irrational,” regardless of whether they are themselves the source of distress. This interaction has led to an overall approach to psychological research and treatment which stigmatizes patients (particularly those on the schizotypal spectrum) while concurrently falling short in developing effective treatments and models due to inherent methodological flaws; despite clear evidence that current taxonomies are unstable and that the assumed biological mechanisms underlying them do not align with their framework. Moreover, given the shared genealogy of both these processes, they are self-reinforcing and inherently perpetuated through the systems and approaches they generated. Without a revolution within the paradigm (systemic schema), psychology as a whole will struggle to fully grasp its subject matter (the psyche). Much as in Piagetian models, it is the failure of schemata to account for experience through assimilation that sparks accommodation. Current evidence from within our paradigm indicates a similar process needs to occur to progress. Thus, developing a full conceptualization of schizotypy requires an act of decentralization and a re-examination of the current body of evidence as a whole if the field is to mature.

In contrast to categorical approaches, current evidence suggests that adopting a cybernetic model better captures the complexity of the phenomena, the etiology of pathological development, and ultimately offers insight into the phenomenological bases of and treatment approaches for the schizotypal population. Briefly, cybernetic models seek to map the behavior of complex self-regulating systems. The mathematician Norbert Wiener defined cybernetics as the study of “control and communication in the animal and the machine.” [46] and noted its applicability to biological systems, computer systems, and broad organizational structures such as governments. What must be understood is that within a cybernetic model, a number of interlocking processes exist within a network of mutually influential relationships. Such systems are reactive and attempt to reach equilibrium through alterations in one or more of their domains. In the case of small disturbances, a cybernetic system may merely make a minor adjustment in one domain to achieve homeostasis; however, in cases where a sufficiently large disruption occurs, the system as a whole may reorganize into an entirely novel point of balance. In such systems, feedback loops between systems are conceptualized; accounting for how over time relatively minor interactions can reinforce and strengthen each other sufficiently to cause such a restructuring. It should be noted the conceptual resonance such a framework has with Piagetian schemata, Kuhnian paradigms, and many psychodynamic conceptualizations of personality development.

With this in mind, the following sections will begin outlining relevant insights gained across a number of disciplines outlining the qualities of the proposed schizotypal population and suggesting the important factors contributing to the development of experiences such as schizophrenia.

### The Schizotypal spectrum within categorical models

While schizophrenia spectrum disorders have been recognized categories of pathology for many decades, the debate about whether there is an underlying genotype or phenotype which preceded each disorder is ongoing. Indeed, there are larger limitations in the assessment of schizophrenia spectrum syndromes than any assessment’s individual construct validity. These are understandable, due to the disorder’s complex etiology and overall institutional focus on diagnosis as a starting point. Given the vast number of contributing factors both preceding and subsequent to formal diagnosis, capturing the most salient dimensions of any particular patient’s experience requires a long list of assessments and extensive clinical interviewing. That is, if there were a healthy population out of which schizophrenia spectrum disorders arise, one cannot know their characteristics except perhaps through post-hoc inference as assessments capture only symptoms of the most extreme pole of disorders. Currently, there is no comprehensive assessment covering all or even most of the domains noted through clinical research and experience. As such, developing an informed treatment plan would demand a complex exploratory phase and numerous specific follow-up assessments to achieve reliable effectiveness; however, given the stereotyped nature of current schizophrenia treatment [1–4], such a comprehensive assessment would likely be too unwieldy for clinical use, or so broad as to merely perpetuate the problem.

Despite this, the schizotypal spectrum exists implicitly as an entire chapter in the DSM (though syndromes are arbitrarily demarcated) in the temporal evolution from brief psychotic disorder through to formal schizophrenia. In contrast, the autism spectrum exists as a single F-code with level of impairment handwritten in (Levels 1–3). At the present moment, the field appears to be quite confused as to how to understand the schizophrenia spectrum. This factor remarkably complicates assessment. Despite diagnostic confusion, known empirical correlates exist with MMPI3 and Rorschach, for example; however, such correlates exist for personality traits [47], Mondal & Kumar, 2021), which may be helpful in diagnosing shizoid PD and schizotypal PD, but less helpful for a brief psychotic episode all the way through to formal schizophrenia. One’s transient state greatly impacts presentation, a second complicating factor of assessment. Thirdly, scales on the MMPI such as Scale 8 (entitled “Schizophrenia” on the MMPI2) and Restructured Clinical Scale 8 (RC8; entitled “Bizarre Ideation”) on the MMPI3 do a fine job gathering data on positive symptoms, as does the Achenbach System of Empirically Based Assessment’s “Thought Problems” subscale [48], however, negative symptoms are easier to overlook and possess a more abstract developmental quality. This is decidedly problematic given the evidence that it is negative symptoms which most influence the etiology and the treatment of schizophrenia spectrum disorders [49–51].

Toward issues around diagnosis the problem is even more obtuse. As the current diagnostic model requires the presentation of 2 or more serious symptoms such as hallucinations or delusions for a significant period of time and persistence of disturbance for six months [8], clinicians are caught in an orientation of triage, approaching the problem after the fact. While the DSM-5TR does imply a manner of progression from brief psychotic disorder to schizophreniform disorder and finally schizophrenia, this interpretation also focuses on the presentation of the most extreme symptoms, creates an observational perspective (altering diagnoses as various milestones are reached), and ultimately fails to properly account for the broad heterogeneity of patient presentation and differential reactions to treatment [52, 53].

It is an essential theoretical assumption that underlies the current paper that these categories more accurately represent extreme presentations along a spectrum of “schizotypy”; essentially, a spectrum which manifests diversity in presentation. Similar models already exist within psychology [54], as does the overall diagnostic mindset (e.g. identifying and treating those on the autism spectrum). It is believed that the spectrum framework better accounts for the heterogeneity of presentation and treatment outcome within the population with implications for more accurate prognosis and effective treatment. This also normalizes and contextualizes the variability and range of symptom expression. Said normalization carries not only ethical implications but also suggests dimensions of treatment that offer increased dignity and resilience to those currently experiencing the spectrum’s most distressing presentations while simultaneously opening avenues for pre-morbid interventions to prevent many otherwise healthy schizotypal individuals from experiencing said distress and its accompanying stigma. Indeed, “because the incontrovertibly psychotic diagnosis of schizophrenia fits people at the disturbed end of the schizoid continuum, and because the behavior of schizoid people can be unconventional, eccentric, or even bizarre, non-schizoid others tend to pathologize those with schizoid dynamics” [55], p. 196). Schizotypes find themselves in a double-bind: those with poor insight often have poor outcomes, and those who possess high insight are frequently besieged with depression, low self-esteem, and suicidality [56]. Thus, developing a comprehensive and destigmatizing model is an essential element in treating the population.

The presence or absence of psychosis is not an appropriate criterion measure of a distinct schizophrenia spectrum condition, nor is it deviant or divergent. Approximately 7% of the general population will have a psychotic experience within their lifetime. Of those 80% will be transitory, with only 7% going on to develop a psychotic disorder [21]. Psychotic experiences are also transdiagnostic and thus may be inappropriately conceptualized as unique to schizophrenia. “It is only when high levels of schizotypy are combined with other aetiological risk factors that an individual may be considered at risk for schizophrenia and other psychotic disorders. According to this perspective, unless high schizotypy is combined with other risk factors, it is considered neutral in regards to psychopathology” [57], as cited in [20].

The overall focus on psychosis (and, its “irrational” positive symptoms) is an axiomatic bias. However, there is empirical and clinical evidence that a population exists which is predisposed to psychotic experience and more likely to do so for much longer periods of time. If true, two questions must be answered. Firstly, what are the qualities which define this population and how do these qualities relate to psychotic experiences? Secondly, what factors (internally and externally) select some members for pathological expression?

## Dimensional models

### Spectra of phenomenology

There is significant evidence supporting a dimensional reframing of psychological disorders [3, 39, 40]. During DSM–5 field trials, 40% of diagnoses did not meet cutoff for acceptable interrater reliability (IRR). Operationalized dimensionally, the same disorders achieved excellent IRR [39]. A dimension, in this context, is a psychological continuum stretching from the average range to extreme expression. It is the individual’s degree along a dimension and his specific dimensional interactions that ultimately lead to the higher order complexes addressed in psychotherapy.

The Hierarchical Taxonomy of Psychopathology (HiTOP) model, for example, describes ascending levels of complexity beginning with dimensions and rising through components, traits, syndromes, subfactors, spectra, and super-spectra. In such a conceptualization, an individual traditionally diagnosed as having depression, anxiety, and an attentional disorder is understood instead as having an interlocking network of specific and interacting anxiety, avoidant, and/or internalizing dimensions. Ultimately, a dimensional framework addresses many of the problems within categorical models, including heterogenous presentation, comorbidity, diagnostic instability, and unstable boundaries with normal psychological functioning, all while having a much stronger empirical basis [39, 40].

In research on schizotypy and psychoticism, strong evidence exists that individuals manifesting these disorders instead represent a small cross-section of a more diverse psychological phenotype within the general population. As was noted, clinically significant psychotic experiences are not uncommon in the general population [21]. In a six-year general population study, it was found that subclinical positive psychotic experiences themselves were insufficient to predict transition into clinical disorder; alternatively, it was the presence and persistence of environmental factors such as childhood trauma, developmental problems and ethnic minority status, as well as severity of secondary distress due to these experiences that best predicted a disorder status [3, 48]. Most individuals with psychotic experiences also carry an additional diagnosis (most often a mood disorder), and the presence of such a disorder is highly predictive of poor prognosis [21]. This is consistent with epidemiological research indicating that the negative symptom dimension (such as poor emotional expression and avolition) is a strong predictor of outcome measures, including the need for treatment at all [58].

As any phenomenology is reactive to its environment, it can be understood how stressful and traumatic experiences can begin altering patterns of cognition and behavior along such dimensional lines. While the more extreme presentations along the schizotypal spectrum (catatonia, flat affect, delusional thinking, etc.) may appear entirely unique, they are not inconsistent with trauma research. Those suffering traumatic or sufficiently stressful experiences often display magical thinking, irrational narratives, affectively-driven reactions incongruous with present reality, and behavioral tendencies towards withdrawal, explosive externalization, and somatic behavior [59, 60]. Moreover, such experiences also create neurological and biological changes quite consistent with those in schizophrenia [61–63]. Thus conceived, even the most extreme presentations within the spectrum can be rooted in explicable and often even beneficial human behaviors and predilections, merely pushed beyond their capacity for stress.

Given the above, there is reason to believe that much of the current conceptualization of schizotypal individuals suffers from a kind of survivorship bias. That is, research is conducted and models are created based on those individuals already in sufficient distress to seek help, and in attempting to reverse-engineer an etiology, the most unusual symptoms are given priority. However, while the presence of positive symptoms such as hallucinations or delusions can certainly be distressing on their own, evidence suggests that these symptoms are acute responses to internal suffering and environmental stressors and rarely, if ever, rise to the level of clinical significance outside of prolonged and unresolved stress [48, 64–66]. From a dimensional perspective, any psychopathology is understood as a dynamic interaction of symptoms influencing each other over time. Thus, in the earliest stages of an “illness,” symptoms are diffuse. Specific syndromes manifest only after prolonged influence and interaction. The specific expressed disorder depends on the nature of the stressors, the developmental stage in which they appeared, and their duration [48, 66]. Equally or more important is the individual’s idiosyncratic response style based on differentiation of dynamics between mental states [48].

To reframe the problem in terms of the overall philosophical blind spots outlined earlier: In trying to understand the nature of schizophrenia so late in its etiology, those elements which seem most alien to our implicit beliefs about mental health are accepted as descriptive of and central to “the problem.” Consequently, we ignore those elements driving the observed symptoms, and subsequent treatment becomes mere management of those symptoms most distressing to norms and caretakers. This may be necessary when a schizotypal individual’s perceptions and thought patterns create distress, isolation, or additional issues; however, it is insufficient to claim that merely subduing these expressions is equivalent to successful treatment, if the underlying sources of stress, maladaptive defenses, and/or relational/attachment experiences remain.

## Personality organization and clinical understandings

In her discussion about schizoid personality structure, McWilliams [55] limned its key traits from a psychodynamic perspective: (1) schizotypes are easily overstimulated and report the experience of their own and others’ affect as overwhelming (p. 198), (2) often perceive the world as threatening to damage or distort their individuality and security; “A deep ambivalence about attachment pervades their subjective life. They crave closeness yet feel the constant threat of engulfment by others; they seek distance to reassure themselves of their safety and separateness yet may complain of alienation and loneliness” (p. 201). (3) As favoring the defense of withdrawal (e.g. into fantasy or physical isolation) while often lacking many of the more common defenses (though projection, introjection, idealization, devaluation and intellectualization are not unheard of); “Under stress, schizoid individuals may withdraw from their own affect as well as from external stimulation, appearing blunted, flat, or inappropriate, often despite showing evidence of heightened attunement to affective messages coming from other” (p. 200). (4) they often speak and act in eccentric and non-conforming ways and may have a natural reliance on metaphor, symbolism, and creative expression when conveying thoughts and experiences; “Even when they see some expediency in fitting in, they tend to feel awkward and even fraudulent making social chitchat or participating in communal forms, regarding them as essentially contrived and artificial” (p. 204).

Applied under a dimensional framework (not incongruous with psychodynamic concepts such as defense mechanisms) the above qualities lead to a probability field of likely dynamics. For example, a person who instinctually withdraws when distressed, and receives little internal reinforcement for casual social interaction, is less likely to develop robust interpersonal skills while being simultaneously forced to understand and manage their powerful affect without guidance or community. If such a person also speaks and acts in an eccentric or unusual way, while maintaining sensitivity to others’ reactions, it is likely they will develop an “othered” conception of self. It is straightforward enough to see a potential for self-reinforcing patterns of pain, expression, social failure, withdrawal, and isolation. If this combines with a penchant for imagistic/symbolic thinking/representation, an entirely separate phenomenological language could begin to develop.

Germane to the larger point is that none of these components are pathological in and of themselves. Indeed, McWilliams argued that most schizoid-organized individuals are quite functional, some even highly so. Although they may be stigmatized and misunderstood (even, and perhaps especially, within the mental health field) due to an unexamined normativity bias [67], an effective clinician should explore both the valid content within their unusual expressions as well as the characterological strengths rather than assuming them to be meaningless, aberrant, or dangerous. In fact, she noted that working with such a client may be quite pleasant as they are often well in tune with their own internal dynamics and how those influence their own experiences and broader environment.

### Personality research and parallels

A third conceptually parallel line of inquiry has been conducted within the frameworks of Five Factor Model of personality theory (FFM) which offers to blend the phenomenological depth of psychodynamic understanding with the scientific rigor of empirical inquiry along dimensional lines. While debates about the structure, components, evolution, and even the ontological nature of human personality are nearly endless, FFM is notable for several reasons. From a broadly conceptual standpoint, FFM stands out in that its development was nearly atheoretical; that is, rather than being reverse-engineered from an existing psychological or culturally instantiated models of human nature, it was instead derived in a bottom-up fashion based on factor-analysis of patterns with linguistic representations. This lends a certain assurance that the model contains fewer a priori assumptions than many of the other popular models espoused. Furthermore, from a more purely empirical perspective, FFM has shown remarkable performance in research settings.

While the initial model was developed through a lexical analysis of English, subsequent studies have been performed utilizing numerous other languages (e.g. Filipino, German, Czech, Dutch, Korean, Hebrew, etc.), which have reasonably confirmed the same five dimensional structure [68–71]. Cross-cultural multivariate behavioral genetic analysis demonstrated that the phenotypic structure of the FFM reflected a universal genetic and environmental structure [72]. Longitudinal studies have shown temporal stability across lifetime as well as the dimensions’ antecedent impact on later psychopathology [73, 74], and FFM research indicates that dimensions such as neuroticism and extraversion are central elements underlying the vast majority of currently designated disorders [75, 76]. This level of construct validity is lacking in current DSM-based personality disorders [77], and as was noted earlier in this paper, is an issue with current disorder constructs categorically.

As with any living model, there are varying ways of dividing and organizing the personality dimensions depending on the area of inquiry. For example, some models explore a construct directly labeled psychoticism, while others do not. Given the modular and hierarchical nature of the model though, it largely avoids the decoherence this diversity might otherwise imply. At core, FFM postulates that personality is composed of an individual’s position along five continuous dimensions: extraversion (sociability or positive affectivity), agreeableness (compassion or cooperation), conscientiousness (diligence or constraint), neuroticism (emotional instability or negative affectivity), and openness (intellect or unconventionality). Depending on the level of analysis, each dimension can be meaningfully decomposed into sub-elements (e.g. conscientiousness may be broken into component parts of orderliness and industriousness) [78] which can then be differentiated further into even more specific facets, behaviors, and tendencies. It is worth noting that it is at this level where the chirality between FFM and dimensional models such as HiTOP comes into focus; they do not neatly superimpose when reflected over each other; FFM being a bottom-up model beginning with foundational tendencies and investigating upwards and outwards, most other dimensional models may be viewed as top-down, beginning with a psychopathological state and deconstructing it into it constituent elements and antecedents. In the latter case, explorations of experiences such as hallucinations tend to cease at the point where hallucination-like experiences do. If (as this paper postulates) such higher-order expressions are emergent properties of entirely benign faculties, then such top-down explorations will have little insight into this non-pathological domain. Where FFM shows the greatest potential as a framework is in its potential for providing phenomenologically causal explanations for behavior rooted in “normal” personality structures while offering broad avenues for research into biological instantiation.

The schizotypal spectrum has been a robust area of interest within FFM research for a number of years. Most consistently, schizotypal individuals score highly on trait neuroticism and low on trait extraversion [79–81]. This is unsurprising in light of previously mentioned clinical profiles and the generally accepted symptoms within current taxonomies, as negative affectivity is found to load onto the former, while detachment loads onto the latter [82]. Additionally, research has indicated that low agreeableness is a factor in positive symptoms [81] and perhaps in manifestations overall [83] and some research has implicated low conscientiousness compared to “healthy” controls [52]. Most contemporary factor research highlights specific subcomponents of each dimension (e.g. the level of trust vs. mistrust within trait agreeableness is often indicated as accounting for much of the variability). However, given the high number and variability of these, a full overview is unwieldy.

Of particular interest within FFM schizotypy research is trait openness. While studies into the personality components of psychopathologies have consistently found meaningful contributions for the first four traits, the data around openness is much more variable. This has led to some speculation that trait openness is functionally dissociable from psychological disorders [84, 85],however, it is consistently found to be one of the best personality markers for those on the schizotypal spectrum [86, 87]. In parallel, it has been shown to carry greater variance with PID-5 Psychoticism [86].

The construct of openness provides perhaps the best theoretical basis within FFM for understanding the positive symptoms associated with schizotypy (e.g. hallucinations, delusions, disordered thoughts/behaviors, etc.). Broadly speaking, openness encompasses intelligence and creativity, or one's interest in ideas and one's interest in aesthetics [86]. It may be meaningfully differentiated into subcomponents such as openness to fantasy, aesthetics, feelings, actions, ideas, and values, and it is tied to scores on measures such as divergent thinking and fantasy-proneness [34, 85]. There is an obvious conceptual link between these facets and many of the positive symptoms of interest; however, much of the research looking to tie the trait to specific symptoms has delivered conflicting results [86]. Some of the conflicting findings may be accounted for by the complex nature of the trait. For example, it has been demonstrated that while interest in aesthetics meaningfully predicts variance in positive symptoms, interest in ideas/intelligence has a negative correlation with the same [86, 88]. This finding is supported in neural modeling research demonstrating that psychoticism, openness, and their shared variance were positively related to coherence in the default network (simulation of experience rather than attention to sensory input) and negatively related to coherence in the frontoparietal cortical network (voluntary control of attention), which have each been tied to psychosis and trait intelligence respectively [88]. Further research has shown that the positive dimension is better captured by measuring the “maladaptive” poles of the traits (i.e. the extreme high and low ends of expression) [83, 87, 89, 90]. Within a cybernetic model, this predictive extreme is precisely what would be expected as such extremity would require equally extreme adaptation to achieve equilibrium. Moreover, with the context of the trauma work cited earlier, one would expect that highly stressful experiences would themselves push the natural pathways of behavior into radical adaptation.

Schizotypal individuals obtain higher scores in divergent thinking [91], a trait linked to openness as well as creative performance generally [9, 87, 92]. Fractional anisotropy measurements of white matter integrity have shown “an apparent overlap in specific white matter architecture underlying the normal variance of divergent thinking, openness, and psychotic-spectrum traits, consistent with the idea of a continuum” [92]. As well, trait openness and creative achievement show a negative correlation with latent inhibition (cognitive shielding from information previously coded as irrelevant), indicating a higher psychological permeability [89], consistent with the noted sensitivity and eccentricity of schizoid individuals within psychodynamic understandings.

FFM research generally finds openness to ideas and openness to aesthetics to be distinct factors. There is evidence for opposing influences between the two factors and positive psychotic symptoms, and correlational data indicates a relationship between the aesthetic/fantasy-prone dimension and schizophrenia spectrum disorders; therefore, a discussion of the psychological concept of aesthetics is relevant.

While a full interrogation of the science of aesthetics is beyond the scope of this paper, contemporary literature highlights some salient points about component experience. Firstly, that aesthetic appreciation derives neither from simple perception nor from straightforward complexity, but instead arises as a higher-order experience comprised of an evaluative dimension (sensory‐motor), a phenomenological/affective dimension (emotion‐valuation), and semantic (meaning‐knowledge) and their neural correlates [93, 94]. Secondly, the appreciation arises from the diversity of sources of information that come into play, and the diversity of ways in which this information can be used, combined, and associated [95]. Thirdly, the aesthetic response can be reflexive and momentary, or manifest in long-lasting mood shifts [95]. Notably, this higher-order and emergent experience goes some way toward accounting for some of the difficulty in measuring motivational patterns in openness [36, 96], which often seek to measure the value of merely novel information without context. As well, to the extent that schizotypal-spectrum experiences load onto openness and are dopaminergic, it dove-tails cleanly with dopamine models understanding the phenomenological function of the neurotransmitter as coding emotional salience [97, 98]. As currently the dopamine hypothesis is one of the leading biological explanations for schizophrenia-spectrum disorders, this begins to offer a more intuitive understanding of such findings.

Comprehensively, this evidence indicates that the aesthetic dimension of openness implicated in schizotypal research is driven by an experience of derived or constructed meaning in complex stimuli. That is, recognizing and associating patterns (across sensory, affective, and cognitive levels) and deriving meaning. This is important as it begins to provide a phenomenological outline with potential neural mechanisms for many of the seemingly more inexplicable traits associated with the spectrum such as delusional thinking, disordered speech, and magical thinking in scientific research, as well as the noted predilection for symbolic and metaphorical understanding and high affective sensitivity in psychodynamic conceptualizations.

## Contemporary approaches

Schizophrenia and related disorders occupy a unique place within the social consciousness. While contemporary discourse around mental health has demystified and destigmatized many disorders, experience of psychosis is rarely included in this trend. Even within the mental health field, individuals experiencing psychotic symptoms are differentially received. Clinicians across disciplines stigmatize patients with schizotypal spectrum syndromes more than patients with other diagnoses [99–101]. “Schizophrenia is one of the most serious and frightening of all mental illnesses. No other disorder arouses as much anxiety in the general public, the media, and doctors” [102], p. 91).

De-stigmatising psychosis as a symptom both separate from schizophrenia proper and “mad” in it’s own right is a hallmark of the Hearing Voices Movement (HVM), which began in the late 1980’s [18, 103]. “Some disability scholars further emphasize the role of ‘madness as testimony’: as Clementine Morrigan explains, so-called symptoms occurring in the wake of trauma may in fact be ‘acts of resistance to violence,’ a means of sounding an alarm that something is very wrong” [18], p. 138).

More research is needed to establish an evidence-base for Hearing Voices Groups (HVGs); however, such psychosocial interventions hold promise, particularly since isolation is often a hallmark of both schizotypy and psychosis. Such group therapies approach treating the voices (auditory hallucinations) as non-pathological and not necessarily a sign that one is mentally ill. Participants in HVGs have reported a sense of higher self-esteem and social competence [104, 105], while those who have learned to form more positive and active relationships with their voices have reported a less negative and sometimes supportive and beneficial relationship with said voices [106–108] Groups are growing, international, and are usually led by a “voice hearer” and a clinical practitioner. Though CBT interventions appear to be the most promising in terms of change mechanisms, more randomized clinical trials are needed [103]. Another psychosocial intervention that may hold promise is the concept of the Phone Pal (Into de Costa, 2020) to combat isolation in those experiencing psychosis. Marriage and family therapy is also effective for treating first-episode psychosis and reducing relapse rates [109].

Read and Dillon [3] utilized a grounded theory approach to collect qualitative data related to identifying effective psychosocial interventions. Researchers found that in cases of first-episode psychosis after which the patient desired to talk about and explore the experience, assisting the patient in such a discussion was therapeutic. Though it may be theoretically “simple,” perhaps it is in the process of relating to a caring and authentic other that one may find and share one’s own voice and begin to consolidate experiences. Such understanding embodies insight, which then results in a patient who “integrates” rather than “seals over” the psychotic experience. “Sealing over is the tendency to dismiss the experience as having little personal relevance, whereas ‘integration’ reflects a curiosity about the experience and its personal significance” [3], p. 180).

Within the Open Dialogue (OD) approach, patients within the population required the use of neuroleptics less frequently and for shorter periods [19]. While more an approach to care than a specific intervention, the hallmark of OD is shared decision- and meaning-making processes, aiming to guarantee both continuity of care and an immediate need-adapted and social network-oriented response. Research into interfamily therapy (which seeks to generate a conversation where experiences can be shared, and emotions can be expressed safely) has indicated lower relapse rates, with fewer psychiatric admissions and of shorter duration among patients during the year of participation [109], while a meta-analysis of 14 studies showed that family intervention in first psychotic episodes led to a 58% reduction in relapse rates, shorter duration of hospitalizations, less severe psychotic symptoms and improved functionality up to 24 months after the intervention [110].

In accounting for trauma within psychosis treatment, post-traumatic growth (PTG) was found to be elicited through narrative interaction with themes of meaning in life, coping self-efficacy and core beliefs; mediating the relationship between total PANSS scores and PTG. Notably, emotional experience was noted as the least frequent facilitator of PTG, casting doubt on the symptom -focused approaches of current treatment [111]. Consistent with this, individuals experiencing psychosis who engaged in poetry as a form of therapy and expression reported greater experiences of integration and acceptance, and overall higher senses of meaning and efficacy, through the practice. It was postulated that such carnivalesque spaces (in which the alternative, transgressive or idiosyncratic are explorable and celebrated) directly supported the wellbeing of the participants [112].

Taken in aggregate, the successes and implications of these approaches lends strong support to the overall premise of the proposed conceptualization. Namely, that the targeted eccentricities of schizotypal individuals are not themselves the issue within the population, but instead it is underlying stress and distress which drives the formation of states such as schizophrenia; and thus must themselves be the target of effective intervention. Moreover, that in reconnecting such individuals with others, providing a voice to meaningfully express their experiences, utilizing their sense of creativity and divergent thinking, and engaging their inner representational dynamic, the suffering experienced by schizotypal individuals can be mitigated without stigma or permanent pharmaceutical interventions.

“A good metaphor for psychotherapy of psychosis could be that it is a form of prayer: striving to bring order out of chaos, helping patients recover confidence in their humanness, seeking something of a resurrection, returning the patient to emotional life from a position of deadness” [3], p. 245).

### Clinical implications

Taken as converging lines of evidence, the aforementioned paradigm allows for a reconceptualization of the psychopathology currently understood as schizophrenia and its related disorders as emergent properties of a particular spectrum of psychological predispositions under stress. Although a dimensional model, it does not adhere to any currently proposed but instead seeks to harmonize the evidence collected across multiple lines of inquiry. With this in mind, we propose a cybernetic model, which accounts not only for the strengths of dimensional templates but provides a means for elucidating the nature of development within and amongst those dimensions over time; offering means of understanding the emergent properties manifested in extreme poles or interactions. Models such as HiTOP are understood as arriving at their traits and dimensions from a predominantly “top-down” approach, working backwards from observed disorders to identify their constituent parts. Models such as FFM are oriented from a predominantly “bottom-up” perspective, and so can better capture what may be meant by normal personality. In so doing, it is proposed that informed clinicians would better understand how a person's natural interest in aesthetics might predispose them to proto-psychotic equilibrated states (thus aiding prognosis and early intervention) while also indicating how one might utilize this trait in strength-based treatment. As FFM already contains frameworks for understanding its dimensions as motivational frames, the component forces driving the homeostatic tendencies within the psyche (which give rise to the defense mechanisms and idiosyncratic feedback loops schizotypal syndromes would likely display) are explicable. While such a view certainly helps to normalize what might otherwise be seen as inexplicable psychosis, it also offers avenues for more robust and bespoke treatment and early identification of at-risk individuals.

At very early ages these individuals would likely have the heritable biological predispositions toward a specific general profile of FFM personality traits; namely some combination of high openness, low extraversion, high neuroticism, and low agreeableness. Probable attributes include high sensitivity to their external environment [55, 113] and relatively socially reserved disposition [55, 114]. Attachment theory research has shown how fundamental habits of behavior within mother-infant dyads create characteristic patterns that reinforce over time (Bowlby, as cited in [115]. Natural inclinations toward introversion and cognitive abstraction are likely to become reinforced by the overall social environment. Additionally, it is feasible that such individuals would be differentially rewarded for information-seeking, complex and conceptual pattern identification, and social interaction. As the individual developed his natural inclinations combined with idiosyncratic environmental patterns, disposition would tend toward the broad personality profiles described in psychodynamic literature, namely introverted, outwardly eccentric, metaphorically and fantasy-oriented, affectively and behaviorally sensitive, and favoring withdrawal when psychologically threatened. They would likely show heightened divergent thinking, be creatively or intellectually motivated, display less regard for social expectations, and show lower levels of trust in others overall.

Where the risk develops is in how these factors can interact under highly stressful and traumatic experiences. A natural tendency to withdraw rather than express leaves a person, particularly a developing child, far more vulnerable to further psychological damage [65, 116]. Childhood trauma victims often develop magical or illogical narratives to conceptualize their experiences while maintaining identity integrity and basic trust. Children who do not externalize distress are far less likely to receive direct help or more mature interpretations from adults in their lives, and thus those beliefs are less likely to be revised. Introverted and eccentric children are predisposed to fewer and less frequent social interactions, yielding a slower growth curve in social competence, thus widening the gap and reinforcing natural tendencies to withdraw. Higher natural neuroticism creates a more sensitive threat-detection response, which coupled with natural distrust, high sensitivity to affect and behavior, divergent thinking, and high internal motivation toward complex pattern resolution, creates a network of feedback loops favoring loose, complex, affectively potent interpretive frameworks built on an internal lexicon to some degree tangential to consensus social understandings. As initially small and disparate behaviors and cognitive tools become habit, they begin to interact and create more complex syndromes based on the individuals’ natural tendencies, their specific environment, and their own phenomenological choices. The specific complexes will be in some ways unique; however, they will follow relatively predictable patterns. Based on the severity and specific combination of these, an individual is then ultimately given a categorical diagnosis of schizophrenia, schizoaffective, etc.

What is principal under this view is that while the traits underlying the more unique features of schizotypal psychosis are involved in the etiology of the disorder, none of them are themselves inherently pathological. Instead, they act as “paths of least resistance,” and in some cases, socio-behavioral risk factors when faced with highly stressful or traumatic experiences. In many ways, the symptoms of delusions, hallucinations, and disordered speech/behavior, etc., would then represent the individual’s greatest psychological strengths pushed well outside of their functional equilibrium and ultimately forced into self-reinforcing feedback loops. However, as it is trauma and emotional pain acting as fuel for these specific symptoms, it is here where treatment ought to focus. Certainly, the presence of psychotic symptoms would necessitate approaches specific to their management and interpretation; however, overall approaches would be formulated much more heavily along trauma recovery lines (i.e. establishing safety, building authentic relationships, reconnecting with the social environment, etc.). Indeed, it is likely that robust and prolonged treatment would need to engage constructively with the individual’s natural areas of strength as part of its process as they will represent that individual’s highest yield sources of positive affect, self-esteem, social recognition, etc. For example, to gain the benefits of greater social engagement, the schizotypal individual must be given the skills to utilize their naturally metaphorical style of communication effectively (rather than pushing them to conform to more traditional social expectations) for the interaction to feel authentic and the sense of connection to be meaningful.

Within a cybernetic model of the human psyche, the state of equilibrium is itself endlessly complex; inasmuch as it requires achieving physiological needs, fulfilling interpersonal needs, maintaining needs around identity and meaning, the solutions to which (in each case) impact one’s ability to do each of the others and more. This is further complicated by the fact that humans are dynamic and goal-oriented creatures, and so this state is itself a moving target constantly informed by experiences and shifting patterns of response. For conceptual purposes only, the specifics will be subsumed into the word equilibrium for now; however, by utilizing this lens, clear bridges can be made between the domains of clinical psychodynamics, personality theory, and biological research. What is frequently discussed under names of defense mechanisms, cognitive distortions, and behavioral patterns, etc., are understood as solutions and corrective measures to achieve this equilibrated state, the specifics of which are shaped by the natural inclinations of the individual (e.g. low extraversion) and their idiosyncratic experiences.

For example, an abused child may develop a narrative of nearly magical self-blame, as their ability to solve the problem of their suffering is nearly zero; however, they must find a logical explanation for their experiences to manage their anxiety, confusion, loss, and pain. Less extreme, a socially anxious person may simply stop engaging with people at all to keep anxiety tolerable. Each person’s specific needs will vary based on their makeup (this is roughly what is called “personality” in FFM), and thus there will be characteristic strategies, obstacles, and areas of flourishing individuals will construct along the way. However, as psyches are permeable structures by any measure, that natural equilibrium point can be moved over a lifetime. Each adaptation creates new forces of its own and must be accounted for by the others, thus necessitating new adaptations. A stressor of sufficient duration or intensity may demand such extreme adjustment that the settling point itself is (more or less) permanently moved. Within the dimensional models such as HiTOP, this is roughly the process by which dimensions impact each other and combine to ultimately create symptoms and syndromes.

In the case of schizotypy, we can highlight some tendencies. Naturally high levels of emotional salience beget heightened need for affect management; as they are likely also to score high in neuroticism, much of this heightened affect is likely to be anxiety-related. Tendencies toward introversion mean fewer opportunities to express internal states or experience other’s internal states. Differential motivation and reward systems create interest toward complex and abstract constructs (ideas, aesthetics, literature, etc.) and favors the individual toward inner worldbuilding over outer worldbuilding. During stressful experiences, natural tendencies to withdraw, to use imagination and abstract problem-solving skills, etc. are favored and likely to become habitual parts of identity. As is with any human trait or capacity, these bring their own sets of challenges and advantages; however, they are themselves neither pathological nor particularly unusual. Nonetheless, under extreme or prolonged stress, his natural tendencies can put the schizotypal person at heightened risk. Tendencies to withdraw mean that they are less likely to receive aid from others, and so prolonged isolated suffering is more likely. Heightened emotional salience means that the likelihood of an emotional overload is increased. Natural strengths in divergent thinking, coupled with tendencies toward abstraction, pattern recognition, and problem solving are likely to leave an individual anxiously searching their environment for explanations for and solutions to their unbearable feelings while receiving very little input from others.

Over time and based on the nature of the psyche-environment interaction, the entire structure of psychic equilibrium can begin moving in profound ways as the individual attempts to use the tools available and the conceptions to which they have experiential access, to navigate the world and manage their own phenomenology. Thus, natural abilities like divergent thinking, or tendencies such as withdrawal into fantasy, begin to themselves become overly stressed and utilized and may themselves become sources of stress as the person moves through life. However, while observing such a mind well into this process (e.g. unequivocal schizophrenia), though its entire makeup may appear fundamentally illogical, it is in fact a complex psychological adaptation to challenges and suffering in life. That is, constructs such as schizophrenia are unstable, heterogenous, and contentious because they are emergent properties within a complex and self-correcting system. While certainly accounting for etiology, heterogeneity, as well as currently clinically unaccounted for though empirically verified biopsychosocial factors, this frame also opens up approaches to treatment that account for differential motivational patterns (as established in FFM research), which indicate potentially effective strength-based modalities for the population.

## Conclusion

The various models, perspectives, and orientations discussed so far represent a wide cross-section of interest into the phenomenon of psychosis and schizophrenia spectrum disorders, as well as personality and psychological research. It is the stance of this paper that these and others represent converging lines of evidence for a schizotypal population naturally occurring within the larger human population overall. Furthermore, this population would span the range from “normal” and “high-functioning” individuals to those experiencing major and prolonged schizophrenic episodes. This schizotypal population would thus be best conceptualized as a cohort “at-risk” of schizotypal psychosis; depending on the number of relevant traits held, their overall intensity, their interactions with each other, and interactions with the environment. The disorders referred to as schizophrenia, schizoaffective, STPD, etc., represent relatively stable emergent states of consciousness appearing as a result of stressful and traumatic experiences within an otherwise healthy population. While their specific presentations may be particularly disorienting and extreme within foundational rationalistic frameworks, they are, in fact, extensions of natural human adaptations under prolonged and/or extreme duress.

Under the proposed conceptualization, a dimensional model similar to HiTOP views the higher-order symptoms accounted for in the DSM as phenomena emergent from specific combinations of more general and mutually influencing sub-traits and behaviors. Rooted in FFM research, this model can be extended beyond simple decomposition of maladaptive traits and defense mechanisms and thus understand how such extreme outcomes arise out of “normal” human personality features while accounting for heritable and biological substrate noted throughout the literature. Functionally, the model reconceptualizes the biological and phenomenological development of more extreme schizotypal presentations as a cybernetic system, in which the ongoing interactions of multiple elements attempting equilibrium to experiences of trauma and stress (whether acute, periodic, or ongoing) arrive at explicable resting states. Thus, disorders such as schizophrenia can be understood as emergent properties of more fundamental systemic interactions rather than discrete disorders in and of themselves. Such a model would account for the clinically significant distinction between those experiencing psychotic episodes and those diagnosed with a schizophrenia-spectrum disorder, as well as the apparent contradiction between the heterogeneity of presentation and the phenotypic resemblance of said disorders.

To the extent that the above is true, this allows not only for a more accurate and tailored understanding of etiology, but also suggests means of risk factor detection early in life and a theoretically sound strengths-based approach to treatment accounting for the underlying affective and characterological engines behind currently targeted symptoms. In so doing, the heterogeneity of traditionally taxonomic disorders is accounted for while offering conceptual bridges between biological, cognitive-behavioral, and psychodynamic understandings of the population and outlining explanatory frameworks for differences between brief psychotic episodes, ongoing and degenerative schizotypal-spectrum disorders, and those cases of total or periodic remission attested to in more culturally diverse literature.

It is the hope of the authors that the proposed understanding of schizotypy as a spectrum rooted in natural and even beneficial psychological functions, and with explicable trauma-driven manifestations, will assist not only in furthering the field’s knowledge of human functioning and treatment of psychosis, but also begin to remove the stigma and aversion which have grown around the concepts. Grounded assessments for early detection will offer incremental validity to a genuinely biopsychosocial approach to research, treatment, and ongoing patient management.

## Data Availability

Not applicable.
